# Illustrative Integration of Patient-Reported Outcomes Version of the Common Terminology Criteria for Adverse Events (PRO-CTCAE) Data in Dose-Cohort Review for a Phase I Clinical Trial

**DOI:** 10.21203/rs.3.rs-10072400/v1

**Published:** 2026-07-02

**Authors:** Carolyn Mead-Harvey, Brie Noble, Claire Yee, Gina Mazza, Blake Langlais, Sheetal Patel, Peter Trask, Lauren Rogak, Ethan Basch, Amylou Dueck, Gita Thanarajasingam

**Affiliations:** Mayo Clinic; Mayo Clinic; Mayo Clinic; Mayo Clinic; Mayo Clinic; Genentech; Genentech; Mayo Clinic; UNC Lineberger Comprehensive Cancer Center; Mayo Clinic; Mayo Clinic

## Abstract

**Background:**

This secondary analysis of a phase I clinical trial illustrates integrated summary and visualization of Common Terminology Criteria for Adverse Events (CTCAE) data and Patient-Reported Outcomes version of the CTCAE (PRO-CTCAE) data at baseline and through the first cycle of treatment for tolerability review during dose-cohort evaluation in a phase I trial.

**Methods:**

This study uses data from a phase I clinical trial of patients with relapsed or refractory acute myeloid leukemia. Through iterative feedback from clinicians and statisticians, we developed a summary of cycle 1 PRO-CTCAE and CTCAE data for the first dose-level cohort. This summary is intended to demonstrate reporting that would be reviewed to inform a decision about proceeding to the next dose level in a dose escalation trial.

**Results:**

Cycle 1 CTCAE and PRO-CTCAE data for 4 patients enrolled in the first dose cohort are summarized as narrative and tabular summaries and visualized using swimmer and butterfly plots. In this cohort, 3/4 (75%) patients had at least one CTCAE grade 3 or higher adverse event, and all 4 (100%) patients had at least one PRO-CTCAE composite score of 3 during cycle 1. All 4 (100%) patients had at least one symptom for which PRO-CTCAE suggested greater symptom burden than was reflected in CTCAE.

**Conclusions:**

In this illustrative summary of a dose cohort, PRO-CTCAE complemented CTCAE reporting by capturing patient-reported symptomatic burden that was not always fully reflected by clinician-graded adverse events. Reviewing PRO-CTCAE and CTCAE data together during dose-cohort evaluation may enrich discussions on safety and tolerability in phase I trials.

## Introduction

Patient-reported outcomes (PROs) are used to measure domains of health-related quality of life, including physical and mental well-being, role function, and symptoms. Although PROs have historically been assessed in later-stage phase III/IV trials, there is increasing research focus on tolerability and treatment-related side effects which are both key endpoints in early phase trials. Work is being done to study how to introduce PROs in phase I trials. FDA guidance has suggested a modular approach to PRO assessment in clinical trials, focusing on measurement of core PROs including symptomatic adverse events.^1^ PROs can function as endpoints for analysis at the end of a trial or can be used for interim analysis decisions such as making dose escalation decisions within a phase I trial design.^2–5^ The OPTIMISE-ROR project has published recommendations for measuring PROs in dose-finding trials, including guidance on core concepts to measure, definition of endpoints by dose-level and time period, and inclusion of PROs in final dose-decision making.^6^ The OPTIMISE-AR project similarly reported approaches for analyzing and visualizing PROs across dose levels (i.e., at final analysis) in early phase dose-finding trials.^7–8^ While experts agree that PROs should and can be used in early phase trials, there is no consensus on when and how to incorporate information from PROs into decision-making. Specifically, the work of Alger et al.^9^ reported that there was no agreement on whether PROs should be used to guide dose decisions at final and/or interim analyses, and no agreement on whether PROs should be used descriptively or for formal decision making.

Phase I trials are designed to evaluate the safety and tolerability of new treatments. Review of adverse events (AEs) experienced by trial participants is a critical aspect of these early phase trials. Current practice for safety and tolerability data collection is for clinicians to evaluate AEs using the Common Terminology Criteria for Adverse Events (CTCAE). Past work has demonstrated that clinicians may miss or underreport symptomatic AEs, including in the phase I setting.^10–11^

The patient-reported complement to the CTCAE is the Patient-Reported Outcomes version of the CTCAE (PRO-CTCAE), an item library of questions for patients to self-report symptomatic AEs in clinical trials, PRO-CTCAE offers a patient-centric method for capturing symptomatic AEs.^12–13^ The PRO-CTCAE library includes questions using patient-friendly terms to assess frequency, severity, and interference of symptomatic AEs on 0–4 verbal descriptor scales (e.g., pain severity is rated by patients as none, mild, moderate, severe, or very severe).

The objective of this secondary analysis was to illustrate a practical approach for summarizing and visualizing PRO-CTCAE and CTCAE data at baseline and through the first cycle of treatment for ongoing tolerability review during dose-cohort evaluation (i.e., at interim analysis) in a phase I trial.

## Methods

This secondary analysis uses data from a phase I clinical trial of patients with relapsed or refractory acute myeloid leukemia (ClinicalTrials.gov: NCT02670044, Registration Date 2016-01-28). Through iterative feedback from team members, which included clinicians and statisticians, we developed an example summary of cycle 1 PRO-CTCAE and CTCAE data for the first dose level cohort of this trial, comprising 4 patients. This summary is intended to be representative of the type of reporting that would be reviewed to make a decision about proceeding to the next dose level in a phase I dose escalation trial. Only data from cycle 1 of the first dose level cohort is summarized, reflective of the real-world situation in which a dose level review is conducted before further data are available.

In this trial, therapy was administered on 28-day cycles. Patients completed PRO-CTCAE items related to 24 selected symptoms (aching muscles, acne/pimples, anxiety, arm or leg swelling, bloating of abdomen, blurry vision, constipation, cough, decreased appetite, diarrhea, difficulty swallowing, dizziness, dry mouth, dry skin, fatigue, headache, itchy skin, mouth or throat sores, nausea, pain, pain in abdomen, problems tasting, shortness of breath, and vomiting), on an electronic device at pre-specified timepoints throughout the study. For this analysis, PRO-CTCAE data from day 1 (prior to treatment start, used as a baseline for analysis purposes), and days 8, 15, and 22 of the first cycle of treatment were utilized. PRO-CTCAE attribute items for each symptom are scaled 0–4; PRO-CTCAE composite scores combine individual attribute item scores for a given symptom into a single score (e.g., combing frequency, severity, and interference scores for pain into a single composite pain score) and are scaled 0–3 to mimic CTCAE grade levels which are mostly limited to a maximum grade of 3 for symptomatic adverse events.^14^

Prevalence of patient-reported symptoms are reported for the overall cohort as counts and percentages of patients with baseline-adjusted score > 0 and ≥ 3 for each PRO-CTCAE item and composite. In baseline-adjustment, the worst score during cycle 1 for each item and composite is reported, if that score is worse than the patient’s baseline score; if the score is the same or better, then a score of zero is reported to avoid misattribution of a symptom present at baseline to study treatment.^15^

For each patient, narrative summaries of post-baseline PRO-CTCAE items of score ≥ 3 and CTCAE items of any grade are presented. PRO-CTCAE baseline scores are given to provide context and evaluate treatment-emergent symptoms.

PRO-CTCAE and CTCAE data for three symptoms (vomiting, nausea, and diarrhea) are visualized using swimmer and butterfly plots. These novel graphical presentations visualize PRO-CTCAE and CTCAE within the same plot, allowing for assimilation of the two measures of patient symptoms.

## Results

We summarized cycle 1 PRO-CTCAE and CTCAE data for the 4 patients enrolled in the first dose cohort. In this cohort, 3/4 (75%) patients had at least one CTCAE grade 3 or higher adverse event, and all 4 (100%) patients had at least one PRO-CTCAE composite score of 3 during cycle 1. All 4 (100%) patients had at least one symptom for which PRO-CTCAE suggested greater symptom burden than was reflected in CTCAE documentation, although the two measures capture related rather than identical constructs.

**Figure 1:**
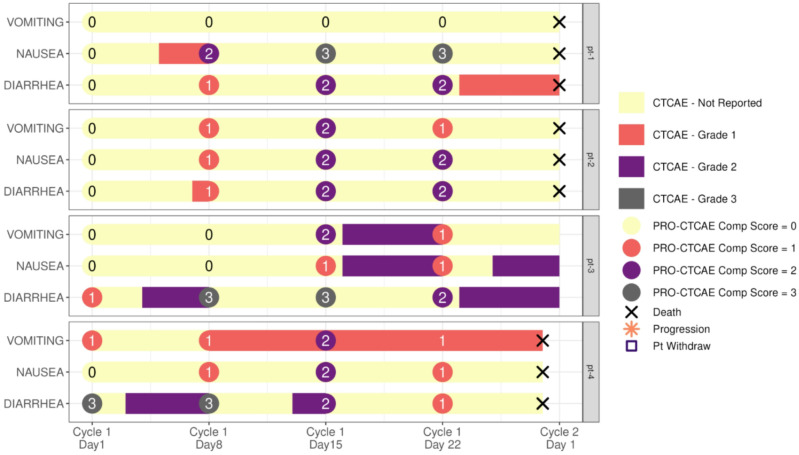
Patient-level CTCAE and PRO-CTCAE for vomiting, nausea, and diarrhea

Patient-level summaries of PRO-CTCAE and CTCAE data are shown in Table 1 and Fig. 1. Table 1 provides an example narrative summary for one patient. In this patient, PRO-CTCAE highlighted several post-baseline symptom worsening events that were not fully reflected in CTCAE documentation, including dry mouth (patient reported a severity score of 3, but no dry mouth adverse events were reported by CTCAE) and diarrhea (patient reported a frequency score of 4, while CTCAE was recorded as grade 2).

Figure 1 displays longitudinal patient-level PRO-CTCAE and CTCAE data for vomiting, nausea, and diarrhea. Within each patient-symptom row, the bar color reflects CTCAE grade and the bar length reflects the adverse-event interval. The color and number within each circle indicate the PRO-CTCAE composite score. Differences between the CTCAE bars and PRO-CTCAE markers were common, which is expected given differences in reporter, scale structure, and assessment timing. For example, in the first represented patient, nausea was graded as CTCAE grade 1 from day 4 to day 8, whereas the PRO-CTCAE composite score was 2 at day 8 and increased to 3 thereafter. Death, progression, or withdrawal events can also be displayed; in this cohort, patients 1, 2, and 4 discontinued study treatment due to death at the end of cycle 1, as indicated by an X in the plot.

At the cohort level, Tables 2 and 3 summarize counts and percentages of patients with baseline-adjusted PRO-CTCAE item and composite scores > 0 and with high symptom burden (item score 3 or higher or composite score of 3). For example, 3/4 (75%) patients had baseline-adjusted diarrhea frequency item scores 3 or higher, suggesting substantial treatment-emergent diarrhea burden in this cohort.

**Figure 2:**
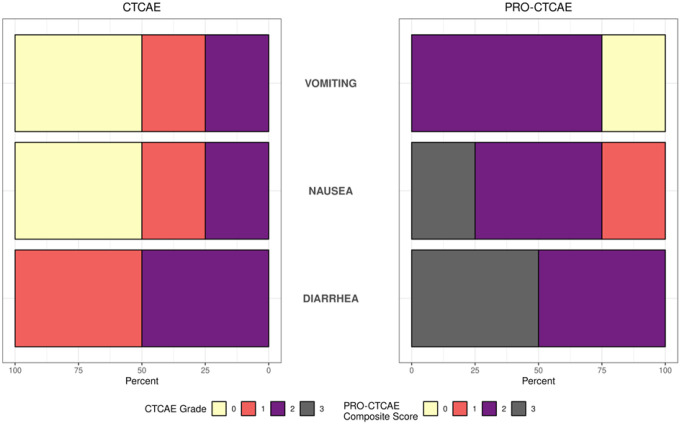
Dose cohort-level CTCAE and PRO-CTCAE for vomiting, nausea, and diarrhea. Data summarizes assessments at cycle 1 day 1, day 8, day 15, and day 22.

Figure 2 compares cohort-level PRO-CTCAE and CTCAE summaries for vomiting, nausea, and diarrhea. Color denotes CTCAE grade on the left and PRO-CTCAE composite score on the right. The top row illustrates cohort-level vomiting results. Based on CTCAE data, 2/4 (50%) patients had any-grade vomiting during cycle 1, including one patient with grade 2 and one patient with grade 1 vomiting. Based on PRO-CTCAE composite scores, 3/4 (75%) patients reported vomiting, all with a maximum composite score of 2.

## Conclusions

In this illustrative cohort, PRO-CTCAE complemented CTCAE reporting by capturing symptomatic burden from the patient perspective that was not always fully reflected in clinician-graded adverse events. Joint review of PRO-CTCAE and CTCAE data during dose-cohort evaluation may enrich discussions of safety and tolerability in phase I trials, but prospective evaluation is needed before such summaries are used to influence formal dose-escalation decisions. This descriptive approach is aligned with the goals of FDA Project Optimus, which emphasize dose optimization by considering safety and tolerability alongside efficacy.^16^ Weekly PRO-CTCAE data collection in the initial cycles was necessary in this trial to comprehensively evaluate patient-reported symptomatic adverse events. This schedule aligns with FDA guidance, which recommends more frequent assessments (e.g., weekly) during the initial treatment cycles.^1^

There is no consensus on appropriate methods for analyzing and visualizing PROs in early phase dose-finding trials.^7–9^ There continues to be growing interest, but the optimal approach to incorporate PRO-CTCAE data in decision-making for phase I trials is still evolving.

Care should be taken to avoid overinterpreting results from small cohorts. While small cohort size may limit ability for formal hypothesis testing or advanced modeling, the nature of a small sample size may open possibilities for more detailed patient-level summarization and visualization.

## Figures and Tables

**Table 1:** Example of a patient-level narrative summary

Overall Summary
Patient 3 completed PRO-CTCAE questionnaires on c1d1, d8, d15, and d22. At baseline, this patient endorsed many symptom attributes at score 1 and 2. During cycle 1, diarrhea frequency reached at score 4 (baseline 2) and three other symptoms reached score 3: dry mouth severity (baseline 0), arm or leg swelling frequency (baseline 2), and decreased appetite severity (baseline 1). In cycle 1, this patient had no grade 3 + AEs.
**Cycle 1 Patient-reported symptoms**^1^ **(score 3+)**
Score 4
Diarrhea frequency (baseline 2)
Score 3
Dry mouth severity (baseline 0)
Arm or leg swelling frequency (baseline 2)
Decreased appetite severity (baseline 1)
**Cycle 1 Adverse Events**^2^ **(all grades)**
Grade 2
Bilirubin total increased
Diarrhea
Nausea
Vomiting
Grade 1
Edema limbs
Hyperphosphatemia
Hypoalbuminemia

1 PRO-CTCAE post-baseline maximum scores by attribute are reported, with baseline score given in parentheses.

2 CTCAE terms and grades are reported.

**Table 2:** Prevalence of baseline-adjusted symptom scores > 0 and ≥ 3 by PRO-CTCAE item

PRO-CTCAE Individual Item Analysis	N	With baseline adjustment > 0	With baseline adjustment ≥ 3
	Overall	n (%)	n (%)
Dry Mouth Severity	4	3 (75%)	2 (50%)
Difficulty Swallowing Severity	4	1 (25%)	0 (0%)
Mouth or Throat Sores Severity	4	1 (25%)	1 (25%)
Mouth or Throat Sores Interference	4	1 (25%)	1 (25%)
Problems Tasting Severity	4	4 (100%)	1 (25%)
Decreased Appetite Severity	4	4 (100%)	2 (50%)
Decreased Appetite Interference	4	4 (100%)	1 (25%)
Nausea Frequency	4	4 (100%)	2 (50%)
Nausea Severity	4	4 (100%)	1 (25%)
Vomiting Frequency	4	3 (75%)	0 (0%)
Vomiting Severity	4	3 (75%)	0 (0%)
Bloating of Abdomen Frequency	4	3 (75%)	1 (25%)
Bloating of Abdomen Severity	4	4 (100%)	0 (0%)
Constipation Severity	4	1 (25%)	0 (0%)
Diarrhea Frequency	4	3 (75%)	3 (75%)
Pain in Abdomen Frequency	4	3 (75%)	1 (25%)
Pain in Abdomen Severity	4	3 (75%)	1 (25%)
Pain in Abdomen Interference	4	3 (75%)	0 (0%)
Shortness of Breath Severity	4	1 (25%)	1 (25%)
Shortness of Breath Interference	4	1 (25%)	1 (25%)
Cough Severity	4	2 (50%)	0 (0%)
Cough Interference	4	1 (25%)	0 (0%)
Arm or Leg Swelling Frequency	4	1 (25%)	1 (25%)
Arm or Leg Swelling Severity	4	0 (0%)	0 (0%)
Arm or Leg Swelling Interference	4	0 (0%)	0 (0%)
Dry Skin Severity	4	2 (50%)	1 (25%)
Acne/Pimples Severity	4	0 (0%)	0 (0%)
Itchy Skin Severity	4	1 (25%)	0 (0%)
Dizziness Severity	4	2 (50%)	0 (0%)
Dizziness Interference	4	2 (50%)	0 (0%)
Blurry Vision Severity	4	2 (50%)	0 (0%)
Blurry Vision Interference	4	1 (25%)	0 (0%)
Pain Frequency	4	1 (25%)	1 (25%)
Pain Severity	4	2 (50%)	0 (0%)
Pain Interference	4	2 (50%)	1 (25%)
Headache Frequency	4	3 (75%)	0 (0%)
Headache Severity	4	2 (50%)	0 (0%)
Headache Interference	4	2 (50%)	0 (0%)
Aching Muscles Frequency	4	1 (25%)	0 (0%)
Aching Muscles Severity	4	1 (25%)	0 (0%)
Aching Muscles Interference	4	1 (25%)	0 (0%)
Fatigue Severity	4	2 (50%)	2 (50%)
Fatigue Interference	4	3 (75%)	2 (50%)
Anxiety Frequency	4	0 (0%)	0 (0%)
Anxiety Severity	4	1 (25%)	0 (0%)
Anxiety Interference	4	0 (0%)	0 (0%)

**Table 3:** Prevalence of baseline-adjusted symptom scores > 0 and = 3 by PRO-CTCAE composite

PRO-CTCAE Composite Item Analysis	N	With baseline adjustment > 0	With baseline adjustment = 3
	Overall	n (%)	n (%)
Dry Mouth	4	3 (75%)	2 (50%)
Difficulty Swallowing	4	1 (25%)	0 (0%)
Mouth or Throat Sores	4	1 (25%)	1 (25%)
Problems Tasting	4	4 (100%)	1 (25%)
Decreased Appetite	4	4 (100%)	1 (25%)
Nausea	4	4 (100%)	1 (25%)
Vomiting	4	3 (75%)	0 (0%)
Bloating of Abdomen	4	4 (100%)	0 (0%)
Constipation	4	1 (25%)	0 (0%)
Diarrhea	4	3 (75%)	1 (25%)
Pain in Abdomen	4	3 (75%)	1 (25%)
Shortness of Breath	4	1 (25%)	1 (25%)
Cough	4	2 (50%)	0 (0%)
Arm or Leg Swelling	4	0 (0%)	0 (0%)
Dry Skin	4	2 (50%)	1 (25%)
Acne/Pimples	4	0 (0%)	0 (0%)
Itchy Skin	4	1 (25%)	0 (0%)
Dizziness	4	2 (50%)	0 (0%)
Blurry Vision	4	2 (50%)	0 (0%)
Pain	4	2 (50%)	1 (25%)
Headache	4	2 (50%)	0 (0%)
Aching Muscles	4	1 (25%)	0 (0%)
Fatigue	4	3 (75%)	2 (50%)
Anxiety	4	1 (25%)	0 (0%)

## Data Availability

The data underlying this clinical trial are not publicly available because they are housed and controlled by Genentech and contain participant-level information subject to privacy, confidentiality, and contractual restrictions. Requests for data access should be directed to Genentech/Roche.

## Abbreviations

PRO-CTCAE: Patient-Reported Outcomes Version of the Common Terminology Criteria for Adverse Events
PROs: Patient reported outcomes
AEs: Adverse Events

## References

[1] US Food and Drug Administration Core patient-reported outcomes in cancer clinical trials: guidance for industry. Accessed 5/11/2026. https://www.fda.gov/media/149994/download

[2] Wages NA, Lin R (2025) Isotonic Phase I cancer clinical trial design utilizing patient-reported outcomes. Stat Biopharm Res 17(1):36–45. 10.1080/19466315.2023.2288013 40842959 PMC12365655

[3] Alger E, Lee SM, Cheung YK, Yap C (2024) U-PRO-CRM: designing patient-centred dose-finding trials with patient-reported outcomes. ESMO Open 9(7):103626. 10.1016/j.esmoop.2024.103626 38968929 PMC11278296

[4] Wages NA, Nelson B, Kharofa J, Meier T (2022) Application of the patient-reported outcomes continual reassessment method to a phase I study of radiotherapy in endometrial cancer. Int J Biostat 19(1):163–176. 10.1515/ijb-2022-0023 36394530 PMC10238853

[5] Lee SM, Lu X, Cheng B (2020) Incorporating patient-reported outcomes in dose-finding clinical trials. Stat Med 39(3):310–325. 10.1002/sim.8402 31797421 PMC8411935

[6] Alger E, Aiyegbusi OL, Dueck AC, Minchom A, Pe M, Peipert JD, Snyder C, Symeonides SN, Wilson R, Basch E, Qiao Y, Bates SE, Bulbeck H, Dean L, Di Maio M, Hansen AR, Kholmanskikh O, Kobayashi K, Landers D, Le Tourneau C, Lee JJ, Ma BBY, Marshall LV, Patel S, Petrie J, Pond GR, Prior K, Rantell KR, Reeve JF, Solovyeva O, Wages NA, Weber HA, Calvert MJ, Yap C (2026) International Consensus-Driven Recommendations for Patient-Reported Outcome Research Objectives in Early Phase Dose-Finding Oncology Trials: OPTIMISE-ROR. J Clin Oncol 44(8):709–719. 10.1200/JCO-25-01625 41576310 PMC12959595

[7] Alger E, Minchom A, Lee Aiyegbusi O, Schipper M, Yap C (2023) Statistical methods and data visualisation of patient-reported outcomes in early phase dose-finding oncology trials: a methodological review. EClinicalMedicine 64:102228. 10.1016/j.eclinm.2023.102228 37781154 PMC10541462

[8] Alger E, Regnault A, Dueck AC, Pe M, Grayling MJ, Calvert MJ, Hansen AR, Kholmanskikh O, Lai-Kwon J, Lee JJ, Minchom A, Qiao Y, Rantell KR, Roydhouse J, Snyder C, Symeonides SN, Wages NA, Wilson R, Yap C (2026) A practical toolkit with recommendations for analysing and visualising patient-reported outcomes in early phase dose-finding oncology trials (OPTIMISE-AR). Lancet Oncol 27(4):e218–e230. 10.1016/S1470-2045(26)00018-5 41861833

[9] Yap C, Lee Aiyegbusi O, Alger E, Basch E, Bell J, Bhatnagar V, Cella D, Collis P, Dueck AC, Gilbert A, Gnanasakthy A, Greystoke A, Hansen AR, Kamudoni P, Kholmanskikh O, King-Kallimanis BL, Krumholz H, Minchom A, O'Connor D, Petrie J, Piccinin C, Rantell KR, Rauz S, Retzer A, Rizk S, Wagner L, Sasseville M, Seymour LK, Weber HA, Wilson R, Calvert M, Peipert JD (2024) Advancing patient-centric care: integrating patient reported outcomes for tolerability assessment in early phase clinical trials - insights from an expert virtual roundtable. EClinicalMedicine 76:102838. 10.1016/j.eclinm.2024.102838 39386161 PMC11462221

[10] Veitch ZW, Shepshelovich D, Gallagher C, Wang L, Abdul Razak AR, Spreafico A, Bedard PL, Siu LL, Minasian L, Hansen AR (2021) Underreporting of Symptomatic Adverse Events in Phase I Clinical Trials. J Natl Cancer Inst 113(8):980–988. 10.1093/jnci/djab015 33616650 PMC8502480

[11] Di Maio M, Gallo C, Leighl NB, Piccirillo MC, Daniele G, Nuzzo F, Gridelli C, Gebbia V, Ciardiello F, De Placido S, Ceribelli A, Favaretto AG, de Matteis A, Feld R, Butts C, Bryce J, Signoriello S, Morabito A, Rocco G, Perrone F (2015) Symptomatic toxicities experienced during anticancer treatment: agreement between patient and physician reporting in three randomized trials. J Clin Oncol 33(8):910–915. 10.1200/JCO.2014.57.9334 25624439

[12] Basch E, Reeve BB, Mitchell SA, Clauser SB, Minasian LM, Dueck AC, Mendoza TR, Hay J, Atkinson TM, Abernethy AP, Bruner DW, Cleeland CS, Sloan JA, Chilukuri R, Baumgartner P, Denicoff A, St Germain D, O'Mara AM, Chen A, Kelaghan J, Bennett AV, Sit L, Rogak L, Barz A, Paul DB, Schrag D (2014) Development of the National Cancer Institute's patient-reported outcomes version of the common terminology criteria for adverse events (PRO-CTCAE). J Natl Cancer Inst 106(9):dju244. 10.1093/jnci/dju244 25265940 PMC4200059

[13] Dueck AC, Mendoza TR, Mitchell SA, Reeve BB, Castro KM, Rogak LJ, Atkinson TM, Bennett AV, Denicoff AM, O'Mara AM, Li Y, Clauser SB, Bryant DM, Bearden JD 3rd, Gillis TA, Harness JK, Siegel RD, Paul DB, Cleeland CS, Schrag D, Sloan JA, Abernethy AP, Bruner DW, Minasian LM, Basch E, National Cancer Institute PRO-CTCAE Study Group (2015) Validity and Reliability of the US National Cancer Institute's Patient-Reported Outcomes Version of the Common Terminology Criteria for Adverse Events (PRO-CTCAE). JAMA Oncol 1(8):1051–1059. 10.1001/jamaoncol.2015.2639 26270597 PMC4857599

[14] Basch E, Becker C, Rogak LJ, Schrag D, Reeve BB, Spears P, Smith ML, Gounder MM, Mahoney MR, Schwartz GK, Bennett AV, Mendoza TR, Cleeland CS, Sloan JA, Bruner DW, Schwab G, Atkinson TM, Thanarajasingam G, Bertagnolli MM, Dueck AC (2021) Composite grading algorithm for the National Cancer Institute's Patient-Reported Outcomes version of the Common Terminology Criteria for Adverse Events (PRO-CTCAE). Clin Trials 18(1):104–114. 10.1177/1740774520975120 33258687 PMC7878323

[15] Dueck AC, Scher HI, Bennett AV, Mazza GL, Thanarajasingam G, Schwab G, Weitzman AL, Rogak LJ, Basch E (2020) Assessment of Adverse Events From the Patient Perspective in a Phase 3 Metastatic Castration-Resistant Prostate Cancer Clinical Trial. JAMA Oncol 6(2):e193332. 10.1001/jamaoncol.2019.3332 31556911 PMC6764147

[16] Friends of Cancer Research Optimizing Dosing in Oncology Drug Development (White Paper). Accessed 5/11/2026. https://friendsofcancerresearch.org/wp-content/uploads/Optimizing_Dosing_in_Oncology_Drug_Development.pdf

